# Psychosocial predictors of eating habits among adults in their mid-30s: The Oslo Youth Study follow-up 1991–1999

**DOI:** 10.1186/1479-5868-2-9

**Published:** 2005-08-02

**Authors:** Elisabeth Kvaavik, Nanna Lien, Grethe S Tell, Knut-Inge Klepp

**Affiliations:** 1 Department of Nutrition, University of Oslo, Norway; 2 Department of Public Health and Primary Health Care, University of Bergen, Norway; 3 Department of Research and Development, Haukeland University Hospital, Bergen, Norway

## Abstract

**Background:**

The predictive value of the psychosocial constructs of Theory of Planned Behaviour (TPB) on subsequent dietary habits has not been previously investigated in a multivariate approach that includes demographic factors and past dietary behaviour among adults. The aim of this study was to investigate to what extent TPB constructs, including intention, attitudes, subjective norms, perceived behavioural control, and perceived social norms, measured at age 25 predicted four eating behaviours (intake of fruits and vegetables, whole grains, total fat and added sugar) eight years later.

**Methods:**

Two hundred and forty men and 279 women that participated in the Oslo Youth Study were followed from 1991 to 1999 (mean age 25 and 33 years, respectively). Questionnaires at baseline (1991) included the constructs of the TPB and dietary habits, and at follow-up (1999) questionnaires included demographic factors and diet. For the assessment of diet, a food frequency questionnaire (FFQ) with a few food items was used at baseline while an extensive semi-quantitative FFQ was used at follow-up.

**Results:**

Among men, attitudes, subjective norms and previous eating behaviour were significant predictors of fruit and vegetable intake, while education and past eating behaviour were predictive of whole grain intake in multivariate analyses predicting dietary intake at follow-up. For women, perceived behavioural control, perceived social norms and past behaviour were predictive of fruit and vegetable intake, while subjective norms, education and past eating behaviour were predictive of whole grain intake. For total fat intake, intention was predictive for men and perceived behavioural control for women. Household income and past consumption of sugar-rich foods were significant predictors of added sugar intake among men, while past intake of sugar-rich foods was a significant predictor of added sugar intake among women.

**Conclusion:**

After adjusting for potential confounding factors, all psychosocial factors assessed among young adults appeared predictive of one or more eating behaviours reported eight years later. Results point to the influence of psychosocial factors on future eating behaviours and the potential for interventions targeting such factors.

## Background

The relationship between psychosocial factors and dietary behaviours over time is not clear [1,2]. With the exception of intervention studies that have used psychosocial models to predict dietary changes among adults over periods ranging from a few weeks to a few years [3-6] only a few studies have used longitudinal designs to investigate the relationship between psychosocial factors and dietary behaviours among adults [7-10]. A study by Armitage and Conner [7] employed the Theory of Planned Behaviour (TPB, including the constructs behavioural intention, attitudes, subjective norms and perceived behavioural control) and reported intentions assessed at baseline to be predictive of dietary behaviour (fat intake) three months later. Conner and colleagues [8] also employed the TPB and found that healthy eating behaviour was predicted from intentions and perceived past behaviour measured six years earlier. Kristal and colleagues [9] applied the Stages of Change Model in their study and implemented a follow-up survey after two years. They found that reduction in fat intake and increase in fruit and vegetable intake were differently predicted by psychosocial factors. Respondents who were in the later stages of change for eating a low fat diet and who read food labels made the largest reductions in fat intake, while changes in fruit and vegetable intakes were small and did not reach significance across psychosocial factors. Patterson and colleagues [10] used the psychosocial factors beliefs, knowledge and perceived norms about diet and cancer to investigate the prediction of dietary change over three years. They found that respondents with a strong belief in the diet-cancer connection and those with knowledge about recommendations regarding diet and cancer reduced their fat intake and increased their fibre intake (knowledge only) more than those with no beliefs and no knowledge about the recommendations. However, food composition knowledge and perceived pressure to eat a healthful diet did not predict changes in fat or fibre intake. Given that the TPB constructs – attitudes toward behaviour, subjective norms, perceived behavioural control and behavioural intention – differ from other models, dietary predictions found with the TPB model can not be directly compared to predictions found via other models. Thus, further investigation is needed to determine the ability of TPB's psychosocial constructs to predict dietary intakes among adults. In addition, dietary habits are known to vary with socioeconomic status, with those in higher social classes having more healthful diets than those in lower social classes [11-15]. The association between social class and diet is also seen in Norway [16,17]. Few studies have investigated long term associations between psychosocial and socioeconomic factors and diet among adult men and women [9,18-21].

A previous cross-sectional analysis of Oslo Youth Study participants (at mean age 25 years) found that components of the TPB accounted for 32% of the variance in behavioural intention to eat healthier food during the four weeks following the survey [22]. The aim of the current study was to utilize a longitudinal design to examine to what extent these TPB constructs measured at age 25 years predicted key elements of eating behaviour eight years later. In particular, we were interested in examining the explanatory power of the psychosocial constructs after adjustment for potential confounders, such as socio-demographic factors and previous eating habits.

## Subjects and methods

### Design and subjects

The Oslo Youth Study was initiated in 1979 with participants in 5^th^–7^th ^grade (mean age 13 years, range 11–16 years) from six schools in Oslo, Norway. The purposes of the study were to obtain epidemiological data on risk factors for cardiovascular disease and cancer and to evaluate the effects of a controlled intervention program to prevent the onset of smoking, increase physical activity and improve eating habits [23]. Participants were invited to take part in a follow-up survey in 1981. In 1991 and 1999, the same subjects, with average ages of 25 and 33 years, respectively, were invited to participate in follow-up studies involving self-administered questionnaires. In 1991, psychosocial constructs taken from the TPB and related to healthy eating were included for the first time. Data from subjects who participated in both the 1991 and 1999 surveys are reported here. In 1991, 706 of 947 eligible subjects participated (74.6%); of these, 526 also participated in 1999. However, as seven participants did not complete the dietary questionnaire in 1999, the final cohort consists of 519 subjects or 73.5% of those eligible in 1991.

As part of the Oslo Youth Study, an intervention program was implemented between the 1979 and 1981 surveys in half of the participating schools. Both students who received the intervention and the controls are included in this study. The intervention is described in detail elsewhere [24].

The Oslo Youth study was approved by the Norwegian Data Inspectorate, as well as the City of Oslo's health authorities.

### Outcome measures – dietary habits

In 1999, participants completed a validated quantitative food frequency questionnaire designed to assess usual diet during the past year [25]. The questionnaire included 180 food items grouped together according to the typical Norwegian diet and meal pattern. Questions were phrased as to tap the usual intake, and both frequencies (10 response alternatives) and amounts (ranging between four and 14 response alternatives dependent on food item) were reported by the participants. Four outcome variables were constructed: 1) *Fruit and vegetable intake *(grams per day, including boiled potatoes in accordance with the Norwegian dietary recommendations [26] and a maximum of 150 grams of orange juice); 2) *Whole grain intake *(grams per day, including whole wheat bread, unsweetened breakfast cereals and oat porridge); 3) *Total fat intake *(percent of total energy intake); and 4) *Added sugar intake *(percent of total energy intake).

### Predictor variables

The constructs from TPB (intention, attitudes, subjective norms and perceived behavioural control with respect to healthy eating) were assessed in 1991 [22]. In the 1991 questionnaire, "healthy food" was loosely defined as "foods low in fat, sugar and salt." Behavioural intention was measured by one question: "How likely is it that you will eat healthier food during the next four weeks?" with response options ranging from (1) "Very unlikely" to (5) "Very likely."

#### Attitudes

Seven beliefs were assessed by a probability scale ranging from (1) "Very unlikely" to (5) "Very likely" (e.g., "If I am eating healthier food the next four weeks my cholesterol level will be reduced"). In addition to the belief regarding reducing cholesterol levels, beliefs about reducing body weight, be more fit, reducing risk of coronary heart disease, looking younger, reducing cancer risk and enjoying food more were assessed. The corresponding outcomes were measured by means of the question "How important is it for you to [e.g., reduce your cholesterol level]?" using a scale ranging from (1) "Not important at all" to (4) "Very important." Each belief item was multiplied by the corresponding outcome item and the products were used to construct an indirect measure of attitude (range: 1–20, Cronbach's α: 0.80).

#### Subjective norms

Normative beliefs were assessed by six items on a scale ranging from (1) "Very unlikely" to (5) "Very likely": "Do you believe that your parents/siblings/friends/partners/physician/co-workers think that you should eat healthier food the next four weeks?" Response alternatives included "I do not know," coded as neutral and "I do not have parents/siblings etc.," coded as missing. Motivation to comply with significant others was measured by the following question: "How important is it for you to comply with...?" on a scale ranging from (1) "Not important at all" to (4) "Very important." The responses to each normative belief were multiplied by the corresponding item for motivation to comply. The products were used to construct a subjective norm scale (range: 1 – 20, Cronbach's α: 0.83).

#### Perceived behavioural control

Perceived behavioural control was assessed by six questions measuring participants' beliefs in eating healthier foods under specific circumstances and two questions measuring participants' beliefs in preparing healthier dishes when busy or when tired. The question "To what extent do you believe you are able to eat healthier food if you are...?" addressed these eight situations, e.g., "with people who eat unhealthy food." In addition, a global question was asked about the extent to which participants felt able to prepare healthier dishes. The response scale ranged from (1) "Very little" to (4) "Very much." The responses to each of the nine beliefs were used to construct a perceived behavioural control scale (range: 1 – 4, Cronbach α: 0.82).

#### Perceived social norms

In addition to the constructs from TPB noted above, perceived social norms were measured by asking participants five questions on how important healthy eating was to their parents, partner, best friend(s), siblings, and co-workers using a scale ranging from (1) "Not important at all" to (4) "Very important." Those who checked "I do not know" were coded neutral and those who checked "I do not have....." were coded as missing. The responses to each of the five questions were used to construct a perceived social norms scale (range: 1 – 4, Cronbach's α: 0.69). For a listing of all items, see Øygard and Rise, 1996 [22].

### Dietary habit score

In 1991, a food frequency questionnaire was used to assess dietary intake. The questionnaire included 30 food items and the five response alternatives regarding frequencies ranged from seldom/never to more than once daily. Information about amounts was not collected, and therefore an assessment of total energy intake was not possible. A *Fruit and vegetable score *was composed of reported intake of fruits, vegetables and orange juice. A *Whole grain score *was composed of reported intake of whole wheat bread and breakfast cereals. A *Fat score *was composed of reported consumption of plant margarine, butter, whole fat milk, French fries/potato chips, meat balls, hamburgers and sausages and, similarly, a *Sugar score *was composed of reported intake of chocolate/sweets, cakes/buns and sugar sweetened, carbonated soft drinks. All scores for single food items ranged from (1) "Eat never or seldom" to (5) "Eat several times daily." A composite score was composed by summing values of single food items included in the specific score and dividing by the number of food items included, resulting in a range from one to five for all four dietary habit scores.

### Demographic variables measured in 1999

Educational attainment reported in 1999 was classified in five categories, ranging from "9 years of elementary/secondary school (or less)" to "More than 4 years at college/university." The household's annual income was reported in six categories ranging from "<NOK 200000" to "≥NOK 600000." If household income was missing, personal income was used. This was the case for four women (two married, one single and one divorced) and for five men (all married). Marital status was classified as "Married or co-habiting" versus "Single," which also included those divorced (n = 18) or widowed (n = 1).

### Statistics

Unpaired t-tests and chi-square tests were used to compare baseline characteristics between follow-up participants and non-participants, and to compare men and women.

Pearson's correlation coefficient was used to investigate bivariate associations between all independent and dependent variables. Linear regression analysis was used to predict eating habits at follow-up. The models were tested for interaction by gender and by intervention/control status in 1979/81. As there was significant interaction with gender, men and women were analyzed separately. There was no interaction with intervention status, thus intervention and control groups were combined in the analyses. In the regression analysis, we entered baseline psychosocial factors in model one, demographic factors in model two and past eating behaviour in model three.

The statistical software package SPSS 11.0 for Windows was used in all analyses.

### Attrition analysis

Of the 706 subjects who participated in 1991, 180 did not participate in 1999. No significant baseline differences were found between those who participated and those who did not in 1999.

## Results

Significant gender differences were observed for baseline dietary and psychosocial factors (Table 1). Compared to men, women reported higher scores on fruit and vegetable intake, attitudes and perceived behavioural control, while men reported higher scores on fat and sugar intake and perceived social norms. At follow-up in 1999, no significant differences existed between men and women regarding the dependent dietary variables (Table 2).

**Table 1:** Psychosocial and dietary factors at baseline (mean age 25 years). The Oslo Youth Study 1991

		Men (n = 238*)	Women (n = 279*)	p-value**
Fruit and vegetable score	Range Mean (SD)	1.0 – 5.0 2.6 (0.8)	1.0 – 5.0 3.0 (0.9)	<0.001
Whole grain score	Range Mean (SD)	1.0 – 5.0 2.5 (1.0)	1.0 – 5.0 2.5 (1.0)	0.925
Fat score	Range Mean (SD)	1.0 – 5.0 2.0 (0.8)	1.0 – 4.3 1.8 (0.7)	<0.001
Sugar score	Range Mean (SD)	1.0 – 5.0 2.1 (0.7)	1.0 – 5.0 2.0 (0.7)	0.004
Attitude	Range Mean (SD)	2.4 – 16.7 8.7 (3.1)	2.4 – 18.9 9.9 (3.1)	<0.001
Subjective norm	Range Mean (SD)	1.2 – 15.0 5.6 (3.1)	1.2 – 18.3 5.7 (3.1)	0.612
Perceived behavioural control	Range Mean (SD)	1.1 – 4.0 2.4 (0.5)	1.2 – 4.0 2.6 (0.5)	<0.001
Perceived social norms	Range Mean (SD)	1.0 – 4.0 2.8 (0.5)	1.0 – 4.0 2.6 (0.5)	0.029

* n differ slightly between different variables due to missing values. ** p-value for difference between men and women.

**Table 2:** Dietary and demographic factors at follow up (mean age 33 years). The Oslo Youth Study 1999.

Variable	Categories	Men (n = 240*)	Women (n = 279*)	p-value**
Fruit and vegetable intake	Grams per day, mean (SD)	335 (192)	367 (213)	0.074
Whole grain intake	Grams per day, mean (SD)	143 (128)	125 (99)	0.070
Total fat intake	Per cent from total energy intake, mean (SD)	31.5 (5.9)	32.1 (5.9)	0.250
Added sugar intake	Per cent from total energy intake, mean (SD)	10.9 (7.7)	10.3 (6.3)	0.403
Education	≤9 years	6.7	5.8	0.004
	10 – 11 years	23.3	11.6	
	12 years	25.4	29.1	
	13 – 17 years	25.4	35.3	
	≥17 years	19.2	18.2	
Household income previous year	<200 000 NOK	8.4	12.7	0.048
	200 000 – 299 000 NOK***	15.8	23.6	
	300 000 – 399 000 NOK	19.2	13.1	
	400 000 – 499 000 NOK	24.8	20.0	
	500 000 – 599 000 NOK	17.9	15.6	
	≥600 000 NOK	13.7	14.9	
Marital status	Married or co-habitant	71.5	73.2	0.696
	Single	28.5	26.8	
Children, number	No children	46.3	30.4	0.002
	1 child	20.8	24.6	
	2 children	25.0	37.0	
	3 children or more	7.9	8.0	

* n differ slightly between different variables due to missing values. ** p-value for difference between men and women. ***NOK = Norwegian krone.

Table 3 presents bivariate correlations between independent variables in 1991 (except dietary scores) and 1999. The correlation coefficients ranged from zero to moderately high. The strongest internal correlations were found among the psychosocial factors, between attitude and subjective norms (0.38 and 0.48 for women and men, respectively), and between attitude and intentions (0.47 and 0.50 for women and men, respectively). For men, the only psychosocial and demographic factors associated with dietary intakes were perceived social norms (with added sugar intake) and education in 1999 (with whole grain intake in 1999). For women, subjective norms, perceived behavioural control and perceived social norms measured in 1991 were associated with two or three of the dietary habits measured in 1999. Furthermore, all demographic factors were associated with fruit and vegetable intake among women, while education in addition were associated with whole grain, fat and sugar intake among women. Bivariate correlation coefficients (Pearson's r) between dietary intake in 1991 and 1999 among men were 0.31 for fruits and vegetables, 0.28 for whole grains, 0.13 for fat and 0.34 for sugar. Corresponding values for women were 0.41, 0.34, 0.17 and 0.30, respectively, and all p-values were <0.001 except for fat intake (p = 0.05 for men and p < 0.01 for women).

**Table 3:** Inter-correlations (Pearson's r) between independent and dependent variables by gender (N≈502). The Oslo Youth Study 1991–99.

Men/Women	1	2	3	4	5	6	7	8	9	10	11	12	13
1) Attitude 1991		0.48***	0.20**	0.23***	0.50***	-0.05	-0.07	0.12	-0.14*	0.08	0.01	0.06	0.04
2) Subjective norms 1991	0.38***		-0.07	0.11	0.31***	-0.11	-0.05	0.04	0.02	-0.09	-0.05	0.08	0.09
3) Perceived behavioural control 1991	0.13*	-0.14*		0.17**	0.28***	0.21**	-0.09	0.08	-0.17**	0.02	0.10	-0.02	-0.12
4) Perceived social norms 1991	0.10	0.05	0.22***		0.11	0.03	-0.13*	0.09	-0.02	0.09	0.03	-0.01	-0.13*
5) Intention 1991	0.47***	0.20*	0.31***	0.11		0.06	0.02	0.06	0.02	0.00	0.08	-0.11	-0.06
6) Education 1999	-0.02	-0.18**	0.14*	0.14*	-0.04		0.21**	-0.09	-0.14*	0.08	0.20**	-0.10	-0.05
7) Household income 1999	-0.11	-0.05	0.08	-0.01	-0.09	0.28***		-0.44***	0.16*	0.05	0.05	0.00	-0.10
8) Marital status 1999	0.04	-0.02	0.03	-0.07	0.02	-0.04	-0.53***		-0.30***	-0.08	0.00	-0.09	0.01
9) Number of children 1999	-0.05	-0.04	0.04	0.08	-0.03	-0.18**	0.10	-0.35***		-0.02	-0.04	0.04	0.10
10) Fruit and vegetable intake 1999	0.05	-0.06	0.27***	0.22***	0.09	0.15*	0.12*	-0.18**	0.13*		0.20**	-0.02	-0.14*
11) Whole grain intake 1999	0.08	-0.19**	0.16**	0.07	0.03	0.25***	0.04	0.03	-0.11	0.21***		-0.22***	-0.22**
12) Total fat intake 1999	0.01	0.08	-0.29***	-0.08	-0.03	-0.15*	-0.11	0.00	0.05	-0.20**	-0.26***		-0.10
13) Added sugar intake 1999	0.05	0.12*	-0.03	-0.12*	0.04	-0.13*	-0.08	-0.02	0.02	-0.12*	-0.12*	-0.07	

Upper half represents men, and lower half represents women. All variables are coded in ascending order except marital status that is coded 1 = married, 2 = single/divorced/widow.

*p < 0.05, **p < 0.01, ***p < 0.001.

Table 4 and 5 present results from multivariate analyses of predictors of dietary habits at follow-up. For men, all significant predictors in model one and two remained significant in the consecutive models. This was, however, not so for women. In model two, education was predictive of women's fruit and vegetable intake, while it lost its significance in model three when adjusting for previous behaviour (Table 4). Subjective norms and perceived social norms appeared predictive of women's intake of added sugar in model one; however, this significance disappeared when adjusting for previous behaviour in model three (Table 5). In the final models for men, attitudes, baseline subjective norms and corresponding eating behaviour at baseline remained significant predictors of intake of fruits and vegetables, while education and corresponding eating behaviour at baseline remained predictive of whole grain intake (Table 4). For women, perceived behavioural control, perceived social norms and corresponding eating behaviour at baseline were predictive of fruit and vegetable intake, while subjective norms and education, in addition to corresponding eating behaviour at baseline, were predictive of whole grain intake in the multivariate models (Table 4). For total fat intake at follow-up, intention and marital status were became significant predictors for men and perceived behavioural control was the only significant predictor for women (Table 5). For added sugar intake, household income and past intake of sugar-rich foods were significant predictors for men (Table 5). For women, only past intake remained a significant predictor of added sugar intake at follow-up (Table 5).

**Table 4:** Baseline (age 25) predictors of fruits and vegetable and whole grain intake at follow up (age 33 years). Multiple linear regression analyses; unstandardized (B) and standardized regression coefficients (β). The Oslo Youth Study 1991 – 1999.

**Dietary habits at follow-up Predictors**	Daily fruits and vegetable intake				Daily whole grain intake			
**Model 1**	Men		Women		Men		Women	

Psychosocial factors at baseline	B	β	B	β	B	β	B	β
Attitude	10.43	0.17*	1.07	0.02	-1.51	-0.04	3.84	0.13
Subjective norm	-12.92	-0.18*	-4.41	-0.05	-2.72	-0.06	-7.91	-0.22**
Perceived behaviour control	-11.48	-0.03	88.07	0.21**	23.68	0.09	22.18	0.12
Perceived social norms	33.52	0.08	79.74	0.18**	5.98	0.02	4.18	0.02
Intention	-5.33	-0.03	4.99	0.02	12.08	0.09	1.57	0.02
R^2^/R^2 ^adjusted, %	3.7/1.6		10.1/8.4		2.1/0.0		7.1/5.4	
**Model 2**								
Model 1 + Demographic factors at follow-up								
Attitude	11.17	0.18*	1.66	0.02	-1.31	-0.03	3.67	0.12
Subjective norm	-12.51	-0.17*	-2.46	-0.03	-1.56	-0.03	-7.41	-0.20**
Perceived behaviour control	-17.02	-0.04	85.70	0.20**	16.62	0.06	18.88	0.10
Perceived social norms	36.35	0.09	66.43	0.15*	2.64	0.01	2.19	0.01
Intention	-6.54	-0.03	7.36	0.02	10.89	0.08	1.55	0.02
Education	9.70	0.06	25.40	0.13*	18.03	0.17*	15.24	0.17*
Household income	2.03	0.02	-4.21	-0.03	1.92	0.02	1.21	0.02
Marital status	-27.58	-0.06	-73.40	-0.15	9.61	0.03	-1.86	-0.01
Children	-4.16	-0.02	17.05	0.08	-0.37	-0.00	-8.30	-0.08
R^2^/R^2 ^adjusted, %	4.7/0.8		14.2/11.2		5.1/1.3		11.8/8.7	
**Model 3**								
Model 2 + baseline eating behaviour								
Attitude	12.40	0.20*	0.91	0.01	-2.42	-0.06	3.85	0.12
Subjective norm	-11.06	-0.15*	0.01	0.00	1.02	0.02	-5.76	-0.16*
Perceived behaviour control	-28.44	-0.07	59.38	0.14*	11.60	0.04	10.72	0.06
Perceived social norms	19.52	0.05	52.97	0.12*	-10.87	-0.04	-6.04	-0.03
Intention	-3.04	-0.02	1.16	0.01	7.21	0.06	-0.79	-0.01
Education	0.08	0.00	13.05	0.07	14.63	0.14*	11.02	0.13*
Household income	-0.98	-0.01	-2.97	-0.02	-1.96	-0.02	0.54	0.01
Marital status	-37.43	-0.09	-56.32	-0.12	17.87	0.06	-1.79	-0.01
Children	-4.73	-0.03	11.99	0.05	0.63	0.01	-8.33	-0.09
Baseline eating behaviour†	75.27	0.31**	79.65	0.32**	31.79	0.25***	30.68	0.31***
R^2^/R^2 ^adjusted, %	13.2/9.3		23.1/20.1		10.7/6.6		20.2/17.1	

Marital status is coded 1 = married, 2 = single/divorced/widow, while all other variables are coded in ascending order. † The corresponding eating behaviour in 1991: fruit and vegetable score in 1991 for fruit and vegetable intake in 1999 (grams per day), whole grain score in 1991 for whole grain intake in 1999 (grams per day), *p < 0.05, **p < 0.01, ***p < 0.001.

**Table 5:** Baseline (age 25) predictors of total fat and added sugar intake at follow up (age 33 years). Multiple linear regression analyses; unstandardized (B) and standardized regression coefficients (β). The Oslo Youth Study 1991 – 1999.

**Dietary habits at follow-up Predictors**	Total fat intake				Added sugar intake			
**Model 1**	Men		Women		Men		Women	

Psychosocial factors at baseline	B	β	B	β	B	β	B	β
Attitude	0.25	0.13	0.06	0.03	0.25	0.10	0.03	0.02
Subjective norm	0.20	0.09	0.05	0.02	0.23	0.08	0.31	0.13*
Perceived behaviour control	0.16	0.01	-3.31	-0.29***	-1.29	-0.08	0.72	0.06
Perceived social norms	-0.34	-0.03	-0.15	-0.01	-2.20	-0.13*	-1.83	-0.14*
Intention	-1.24	-0.21**	0.14	0.02	-0.78	-0.10	-0.16	-0.03
R^2^/R^2 ^adjusted, %	4.1/2.0		8.4/6.6		4.3/2.2		3.3/1.5	
**Model 2**								
Model 1 + Demographic factors at follow-up								
Attitude	0.27	0.14	0.04	0.02	0.30	0.12	0.02	0.01
Subjective norm	0.18	0.08	0.03	0.02	0.20	0.07	0.28	0.12
Perceived behaviour control	0.36	0.03	-3.06	-0.27***	-1.31	-0.08	0.98	0.08
Perceived social norms	-0.20	-0.02	-0.37	-0.03	-2.53	-0.15*	-1.97	-0.15*
Intention	-1.23	-0.21**	0.05	0.01	-0.83	-0.11	-0.18	-0.03
Education	-0.43	-0.09	-0.27	-0.05	0.28	0.04	-0.41	-0.07
Household income	-0.16	-0.04	-0.42	-0.12	-0.77	-0.15*	-0.27	-0.07
Marital status	-1.97	-0.15	-0.76	-0.06	-0.07	-0.00	-0.63	-0.04
Children	-0.01	0.00	0.41	0.04	0.86	0.11	0.08	0.01
R^2^/R^2 ^adjusted, %	6.7/2.9		10.2/7.1		7.0/3.2		4.6/1.2	
**Model 3**								
Model 2 + baseline eating behaviour								
Attitude	0.23	0.12	0.05	0.03	0.13	0.05	-0.02	-0.01
Subjective norm	0.17	0.08	0.03	0.01	0.07	0.02	0.16	0.07
Perceived behaviour control	0.41	0.03	-2.91	-0.25***	-1.45	-0.09	1.24	0.10
Perceived social norms	-0.31	-0.03	-0.29	-0.02	-2.30	-0.14*	-1.58	-0.12
Intention	-1.16	-0.20*	0.09	0.02	-0.23	-0.03	0.15	0.02
Education	-0.43	-0.09	-0.27	-0.05	0.74	0.12	-0.30	-0.05
Household income	-0.19	0.05	-0.37	-0.10	-0.89	-0.17*	-0.26	-0.07
Marital status	-2.16	-0.17*	-0.64	-0.05	-0.65	-0.04	-0.90	-0.06
Children	-0.06	-0.01	0.22	0.04	0.42	0.06	0.0	0.00
Baseline eating behaviour†	0.92	0.12	0.56	0.07	3.65	0.35***	2.34	0.26***
R^2^/R^2 ^adjusted, %	8.0/3.9		10.7/7.1		17.6/13.9		10.6/7.1	

Marital status is coded 1 = married, 2 = single/divorced/widow, while all other variables are coded in ascending order. † The corresponding eating behaviour in 1991: fat score in 1991 for total fat intake in 1999 (per cent of energy from total fat), sugar score in 1991 for added sugar intake in 1999 (per cent of energy from added sugar), *p < 0.05, **p < 0.01, ***p < 0.001.

## Discussion

Results of this study are an important addition to the literature on psychosocial predictors of eating behaviour. By employing a longitudinal design and adjusting for socio-demographic confounders, as well as previous eating behaviour, we found that attitudes, subjective norms, perceived behavioural control, perceived social norms and intention to eat healthier food the next four weeks emerged as significant predictors of one or several eating behaviours eight years later. Of the socio-demographic factors, only education was positively associated with healthy eating for both sexes, while a higher income was associated with a low sugar intake and to be single or divorced was associated with a lower fat intake among men. Overall, the factors examined accounted for 4% to 20 % of the variation in follow-up eating behaviour.

### Fruit and vegetable intake

For men, neither subjective norms nor attitude was significantly correlated with fruit and vegetable intake in bivariate analyses. However, in multivariate analyses, these factors appeared to be significantly associated with the intake of fruits and vegetables at follow-up. Because no significant associations existed between psychosocial factors and fruit and vegetable intake among men in bivariate analyses, multivariate associations might be artefacts, but they could also be a suppression phenomenon, see under *Internal correlations *below. Perceived behavioural control and perceived social norms measured at baseline remained predictive of women's intake of fruits and vegetables. In the 1991 survey, perceived social norms were found to be predictive of healthy eating [27]. In 1999, subjective norms were negatively associated with intake of fruits and vegetables because of the way the question was asked: "Do you believe that your parents, etc. think that you should eat *healthier *food the next four weeks?" Thus, those already having a healthy diet most likely did not believe their significant others expected them to eat healthier food. Conner and colleagues [8] investigated the longitudinal relationship between the constructs of TPB assessed at two time points six months apart (time 1 (T1) and time 2 (T2)) and eating behaviour assessed six years after T2. They found that intentions and the interaction between intentions and intention stability (defined as stability in intention between T1 and T2 six years prior to the last follow-up in their study) and between perceived past behaviour and intention stability were predictive of fruit and vegetable intake six years later. In our study, the constructs of TPB were measured only at age 25; hence, we can not address the stability of the psychosocial constructs. In an earlier publication by Conner and colleagues [28], they concluded that both stability in intentions and perceived behavioural control were important for future dietary behaviour. Our study adds to previous research in that perceived behavioural control, as well as perceived social norms among women measured once several years prior to dietary behaviour assessment, appear to be predictive of dietary behaviour.

Social class and family situation are factors shown to be predictive of dietary habits [15,29-31]. However, none of the socio-demographic factors in our study emerged as predictors of fruit and vegetable intake when we adjusted for past behaviour. This lack of an association may indicate that the association is mediated through other variables, such as dietary habits and psychosocial factors.

### Whole grain intake

Education and previous behaviour remained significant predictors of whole grain intake among men and women in multivariate analyses, while the subjective norms construct was significant only for women. This is consistent with previous findings showing that a higher social class is associated with higher rates of consumption of whole grain foods [14,32]. As for fruit and vegetable intake, the subjective norms construct was negatively associated with whole grain intake. Patterson and colleagues [10] did not find that perceived norms, which resembled subjective norms in our study, explained fibre intake three years later.

### Fat intake

Intention to eat healthier food measured at baseline was significantly and negatively associated with men's fat intake at follow up, even after adjusting for past behaviour. Perceived behavioural control remained a significant predictor of fat intake among women in multiple regression analyses. This is in contrast to the findings of Conner and colleagues, who found that intentions and the interaction between intentions and intention stability were the only significant predictors of fat intake six years later [8]. While Conner and colleagues' study included both genders, women constituted 83% of their sample. Among men in our study, marital status appeared to be a significant predictor of fat intake; to be single/divorced was associated with a lower fat intake when adjusted for psychosocial factors and past behaviour. This is in contrast to a Finnish study finding that married men and women had diets more in line with the dietary guidelines than not married men and women [15]. We have no explanation for our finding, and it may also be spurious.

### Sugar intake

In the final model, perceived social norms and household income were predictive of men's sugar intake. In a previous report from the Oslo Youth Study, perceived social norms, represented by partners, were also predictive of healthy eating when adjusted for education [27]. For women, perceived social norms were predictive of sugar intake; however, when we included past behaviour in the model, past behaviour was the only factor that was predictive of women's sugar intake. A cross-sectional study by Grogan and colleagues examined gender differences in attitudes and behaviour using the Theory of Reasoned Action regarding eating sweet snacks [33]. The authors found that perceived social pressure and attitudes toward sweet snacks were associated with women's intentions to eat sweet snacks, while only attitudes were associated with men's intentions. Both men's and women's intentions were associated with reported intake of sweet snacks. However, the perceived social pressure construct in that study resembled the subjective norms construct in our study, and in that respect, our findings were similar. However, in our study, subjective norms lost its significance in predicting sugar intake when we included demographic factors and past intake of food high in sugar in the models.

### Past eating behaviour

Scores representing past behaviour were predictive of intake of all dependent dietary measures for both sexes, with the exception of fat. Few studies have investigated the stability of nutrient intakes [34] and eating habits [35] during adult years. A study by Mulder and colleagues [35] on the stability of lifestyle behaviour over four years among adult men 30 – 39 years of age at baseline found the correlation coefficient between the dietary scores (including meal pattern, sweet and salty snacks, fruit and attitudes toward eating fat and fibre) at time one and time two to be 0.57. Our findings indicate that past habits are important predictors of current habits even when adjusting for socio-demographic and psychosocial factors and when taking into account that a different measure to assess food intake was used at baseline of the study. Conner and Armitage [36] have proposed that past behaviour may predict future behaviour as a moderator of the relationship between the TPB variables, as a source of information and as a mediator of TPB variables. They argue that there are good reasons to incorporate frequency of past behaviour as predictors of current behaviour in the TPB alongside intentions and perceived behavioural control. The results of our study support the view that past behaviour has an independent predictive value of fruit and vegetable intake, whole grain intake and sugar intake when taking the TPB constructs into account. Previous studies have shown that past behaviours were predictive of future behaviours independent of intentions, attitudes, norms and perceived behavioural control (PBC) [37]. Conner and Armitage [36] reported that after accounting for PBC and intentions, past behaviour, on average, could explain 13% (3% to 28%) of the observed variance in behaviour. In our study, an additional 6.2% on average (ranging from 0.5% to 10.6%) of variance in behaviour was explained by past behaviour beyond what was explained by the TPB variables and demographic factors. Given that the TPB is not often used to investigate dietary behaviours, comparing the explained variance between studies is difficult. The total explained variance in dietary habits in our study is, however, comparable to findings reported by Conner and colleagues [8]. The low explained variances found in our study and in other studies investigating psychosocial and demographic factors' prediction of dietary habits point to other variables having impact on dietary habits. Such factors have not been examined in this study, but physical environment [38,39], as well as taste, cost and convenience have been proposed as important to dietary behaviours [40,41].

### Gender differences

Dietary differences between men and women have previously been demonstrated, with women generally reporting healthier eating habits than those reported by men [42]. Our results at baseline agree with this finding, as women had higher scores on fruit and vegetable intake and lower scores on fat and sugar intake compared to scores among men. However, at follow-up we observed no statistically significant differences between men and women's dietary intakes. In a representative and random sample of Norwegians 16 to 79 years of age in 1993 that applied the same method as the 1999 follow up of the Oslo Youth Study, researchers reported that women had higher intakes of vegetables and of fruit and berries compared to men, while men had higher intakes of cereals compared to women [43]. Also, fat and sugar intake differed between men and women in the previous study, while in our study the differences were not statistically significant. Even though most findings from our study showed the same patterns as in previous studies, differences between men and women did not reach statistical significance. This may be due to differing age groups and places of residence, as these factors also influence dietary intakes assessed with this method [44].

Gender differences in the TPB constructs regarding healthier eating have not been reported previously, but a study applying the Theory of Reasoned Action found women were under more social pressure not to eat sweet snacks than were men [33]. Barker and colleagues demonstrated that fat-phobic and fibre-philic attitudes were more prevalent among women, and that fat-phobic attitudes were inversely related to fat intake among women, but not among men [45]. A Norwegian study showed that, compared to men, women were more prone to consider foods that were in accordance with dietary recommendations as healthy, and less prone to consider fat- and protein-rich foods as healthy [46]. However, the gender difference disappeared when including "trust in experts" in the model, indicating that women's higher trust in experts might be one reason they ranked fish, fruits, vegetables and potatoes as healthy foods. This is similar to Grogan and colleagues' findings that women felt more pressure from health experts than did men to avoid eating sweet snacks [33]. Our results support this finding by indicating that men and women might use different psychosocial bases to carry out certain behaviours, but as we can see no certain patterns regarding gender, we can not conclude about how TPB constructs predict men's and women's eating behaviour differently.

### Limitations

The relatively high attrition rate in this study is comparable to attrition rates found in previous longitudinal studies with similar follow-up periods [47,48]. There were, however, no baseline differences between participants and drop-outs, and we do not think that the attrition seen in this study is a threat to the validity of the observed prospective relationships.

The psychosocial factors in this study were constructed around healthier eating defined as "food low in fat, sugar and salt." Being aware that fruit, vegetables and whole meal bread was not included in the definition of a healthy diet in the 1991 survey is important. In 1991, the dietary focus in Norwegian society, as well as in the Oslo Youth Study, was on reducing the intake of fat and salt. The dietary focus in 1991 influenced the way the TPB questions were phrased and this might have influenced the observed associations between psychosocial factors and dietary habits. Baranowski and colleagues [1] claim that the influence on dietary habits varies by foods, and that the predictive value of TPB appears to be higher in predicting intake of a single food item or narrow categories of foods. Also the variability in measurement may play a role in the prediction of, for instance, fat eating patterns [1], which can be measured by means of total fat intake in grams per day, as per cent of total energy from fat or as foods high in fat. All this will contribute to differing prediction of different behaviours by psychosocial factors.

The time period for which intentions and attitudes toward healthier eating was applied at baseline in this study was "the next four weeks." It is therefore remarkable that the constructs of TPB contributed to the prediction of eating habits assessed eight years later. Results might point to the stability of the underlying psychosocial constructs. These findings are in agreement with previous studies investigating the stability of TPB constructs over time and as predictors of food choice over time [7,8].

The methodology of assessing diet in 1991 and 1999 differed. The dietary method used in 1999 made it possible to compute total intake of energy, macro- and micronutrients, while the questionnaire used in 1991 only enabled assessment of intake frequencies. Prediction of single nutrients in 1999 by means of previous intake was difficult as we did not have measures of the same nutrient eight years earlier. However, the single food items measured in 1991 are good sources for the particular 1999 nutrients [49]. Despite the differing methods used to assess dietary intakes, past behaviour was predictive of current behaviour for all items except fat intake, indicating a high degree of stability in dietary habits. Dietary scores composed of intake of specific food items and nutrients are shown to be valid for evaluating diet quality among adults [50,51].

### Internal Correlations

In multivariate analyses of prediction of fruit and vegetable for men, the high correlations between subjective norms and attitudes might explain why variables not significant in bivariate analyses became significantly associated with the diet under investigation in multivariate analyses. Subjective norms might act as suppressors on attitudes, and vice versa, in bivariate analyses between each of these constructs and the intake of fruit and vegetables (negative confounding). However, in the multivariate analyses, these constructs will be mutually adjusted and the association between each of them and fruit and vegetable intake will become significant. However, the observed associations between attitude, subjective norms and fruit and vegetable intake among men in the multivariate analyses might also be an artefact. The other modest correlations between independent variables in this study are not regarded as a threat to the validity of the results.

## Conclusion

Despite the psychosocial predictor variables investigated in this study being operationalized in terms of predicting healthy eating *four weeks later*, attitudes, subjective norms, perceived behavioural control, intentions and perceived social norms all appeared predictive of one or more specific eating behaviours reported eight years later. This was the case even after adjusting for demographic factors and past corresponding eating behaviour. Results point to the influence of psychosocial factors on future eating behaviours among adults and the potential for interventions targeting such factors on future behaviours.

Future research should focus on further development of appropriate assessment tools for psychosocial constructs, whether such constructs are stable over time and applying parallel measures of dietary intakes over time among representative samples of adults.

## Competing interests

The author(s) declare that they have no competing interests.

## Authors' contributions

EK was responsible for the formulation of the research question, the data analyses and writing the paper. NL contributed to formulate the research question and assisted with data analyses and writing the paper. GST and KIK were responsible for the design of the overall study, data collection and writing the paper. KIK contributed to formulating the specific research question and supervised data analyses. All authors have read and approved the final manuscript.
